# Assessing the influencing factors of out-of-pocket costs on tuberculosis in Sichuan Province: a cross-sectional study

**DOI:** 10.1186/s12889-023-16180-y

**Published:** 2023-07-19

**Authors:** Lan Xia, Lijie Gao, Yin Zhong, Ya Wu, Jinge He, Fengjuan Zou, Ronghua Jian, Sujian Xia, Chuang Chen, Sui Zhu

**Affiliations:** 1 Department of Tuberculosis, Sichuan Provincial Center for Disease Control and Prevention, No.6 Middle School Road, Wuhou District, Chengdu, 610041 Sichuan Province China; 2 Department of Epidemiology and Statistics, School of Medicine, Jinan University, Guangzhou, 510632 China

**Keywords:** Tuberculosis, Out-of-pocket, Total costs, Risk factor

## Abstract

**Background:**

Although diagnosis and treatment services for tuberculosis (TB) are provided free of charge in most countries, direct non-medical and indirect costs due to absenteeism, also place a significant burden on patients and their families. Sichuan Province has the second highest incidence of TB in China, with an incidence of approximately 100 cases per 100 000 people. However, there are limited research on out-of-pocket expenditure (OOPE) and its influencing factors in TB patients in Sichuan Province.

**Methods:**

A retrospective cross-sectional study was conducted on TB patients in designated medical institutions for TB in Sichuan Province from 2017-2021. A face-to-face questionnaire was conducted to obtain the information related to hospitalization of patients, and the multi-level regression model was used to analyse the factors that influence OOPE and total out-of-pocket expenditure (TOOPE) of TB patients.

**Results:**

A total of 2644 patients were investigated, and 74.24% of TB patients and their families experienced catastrophic total costs due to TB. The median total cost was 9223.37 CNY (1429.98 USD), in which the median direct and indirect costs of TB patients were 10185.00 CNY (1579.07 USD) and 2400.00 CNY (372.09 USD), respectively, and indirect costs contributed to 43% of total costs. The median OOPE and TOOPE costs were 6024.00 CNY (933.95 USD) and 11890.50 CNY (1843.49 USD), respectively. OOPE and TOOPE had common influencing factors including whether the patient's family had four or more members, a history of hospitalization, combination with other types of TB, the number of visits before diagnosis, and co-occurrence with chronic disease.

**Conclusions:**

The OOPE and TOOPE for TB patients and their families in Sichuan Province are still heavy. In the long run, it is necessary to strengthen education and awareness campaigns on TB related knowledge, disseminate basic medical knowledge to the public, improve healthcare-seeking behavior, and enhance the healthcare infrastructure to improve the accuracy of TB diagnosis and reduce the significant OOPE and TOOPE faced by TB patients and their families in Sichuan Province.

**Supplementary Information:**

The online version contains supplementary material available at 10.1186/s12889-023-16180-y.

## Introduction

Although there has been progress in tuberculosis (TB) control over the past few decades, TB remains a huge burden of morbidity and mortality worldwide [1, 2]. The World Health Organization (WHO)' Strategy to End TB' sets ambitious goals for 2025, including a 50% reduction in TB incidence and a 75% reduction in TB deaths compared with 2015, and households not affected by TB face catastrophic costs [1, 3]. According to the WHO's latest Global TB report, we can note that the trajectory of the global TB epidemic presents a rather grim picture and that all of the United Nations General Assembly targets are seriously off track [4]. As a result, the annual decline in TB incidence has almost stalled in recent years, and for the first time in nine years, the estimated number of TB deaths has increased [5]. The disruption of TB services due to the COVID-19 pandemic contributes to this result, but it also exposes the vulnerability of global TB services, leaving more TB patients facing a significant economic burden [5].

Poverty is not only a risk factor for TB but also one of the main consequences of TB [6]. Although TB diagnosis and treatment services are free in most countries [7–10], direct non-medical costs such as nutrition and accommodation during TB treatment, as well as indirect costs due to absenteeism also place a significant burden on patients and their families [11]. A study in India concluded that 54.5% of the total economic burden was indirect costs and 45.5% was direct costs, in which the direct non-medical cost constituted 45% [12]. At the same time, a study in Tanzania showed that 85.9% of TB households were economically strained due to TB, and 32.6% of income loss was related to nutritional supplements [7].

Like many other TB high-burden countries, China's budget for TB prevention and control has increased year by year to reduce the economic burden of TB households. However, the economic burden on TB patients is still heavy [13]. A study in Chongqing Municipality showed that the total cost per person for TB treatment reached 14,156.8 CNY, and 80.98% of the patients had experienced catastrophic total costs due to TB [14]. China has the second highest number of TB cases in the world, with an estimated incidence rate of 59/100,000 in 2020 [3]. The Chinese government has been providing free treatment to TB patients under the Directly Observed Treatment Short-course (DOTS) programme, on the recommendation of the WHO [15]. However, this service only covers the cost of direct diagnostic tests and first-line anti-TB drugs, without other related expenses, such as direct non-medical costs and indirect costs which are important issues in TB control [11]. Sichuan Province, located in southwest China, has a highly ethnically diverse population. The economic development level of Sichuan Province has been in the middle class in China, and the proportion of poor people is relatively high [16]. Moreover, the incidence of TB in Sichuan province is at a high level [17], ranked as the second highest incidence of TB in China, with an incidence rate of approximately100 cases per 100,000 people [18]. However, there is relatively scarce research on the economic burden of TB patients in Sichuan Province and its influencing factors.

At present, the current research mostly focuses on out-of-pocket expenditure (OOPE) for TB patients, ignoring the indirect costs caused by the absence of work [9, 19–21]. Meanwhile, studies for risk factors affecting the OOPE of TB are mostly limited to the level of patients, such as age, sex, and occupation, et al, ignoring the possible clustering of patients in different cities [7, 9, 22]. The multi-level model has been widely used in the study of various influencing factors, mainly applied to data with hierarchical or nested structures [23, 24]. The main feature of the data is that the distribution among the survey objects is not independent, but there is a certain degree of aggregation, such as between cities, schools, and hospitals [9, 19–21, 25].

Therefore, we are interested not only in OOPE but also in the total out-of-pocket expenditure (TOOPE) of TB in Sichuan Province, and costs quantify the economic burden in our paper. We aimed to 1) study the OOPE and TOOPE of TB patients in Sichuan Province from 2017-2021, 2) and further assess the influencing factors of OOPE and TOOPE using two-level linear regression models, which could provide information to reduce the burden on patients and improve access to TB treatment.

## Methods

### Study design

A retrospective cross-sectional study was conducted in 25 designated TB medical institutions in Sichuan Province, which were randomly selected. Patients who met the inclusion and exclusion criteria from 2017 to 2021 were surveyed. Inclusion criteria: 1) The diagnostic criteria for pulmonary TB was WS288-2017 [26]; 2) TB patients under treatment for more than two weeks; 3) Patients can express clearly and have the ability to accept questionnaire survey. Exclusion criteria: 1) Patients with TB other than those with pulmonary TB (such as bone TB, intestinal TB, and lymph node TB); 2) Patients with drug-resistant TB (mono-drug resistant TB, multi-drug resistant TB). This survey mainly collected information about the economic burden faced by TB patients and their families. This study was approved by the Ethics Committee of Sichuan Center for Disease Control and Prevention (SCCDCIRB NO.2022-011). All participants were required to obtain informed consent prior to the investigation.

### Study participants

This study used a completely random sampling method to randomly select medical institutions in the National TB Control Program (NTP) network in Sichuan Province. A total of 6 designated provincial/municipal-level NTP network medical institutions and 19 county-level NTP network medical institutions were selected, and each prefecture-level city ensured that at least one NTP network medical institution was selected (Fig. S1).

The sample size of this study was calculated as follows:

$$N={\left(\frac{{u}_{a/2}\sigma }{\delta }\right)}^{2}$$

In the formula, *N* is the sample size, *u*_α/2_ is the significance test statistic, that is, the *u* value corresponding to the test level of *α*; *σ* is the standard deviation of economic costs; *δ* is the allowable error, in this study, *α* is 0.05 and *u*_α/2_=1.96; *σ*=11247.4 CNY through a literature review [14]; *δ*=500 and *N*≈1944. A non-response rate of 15% was assumed in this study, requiring a minimum of 2236 TB patients to be investigated.

### Data collection

A self-made structured questionnaire was administered to each TB patient to collect basic information face to face, and their corresponding electronic medical records were obtained from the hospital information system. The following information was obtained: 1) demographic and socioeconomic information, including gender, age, education level, marital status, registered permanent residence, occupation, family size, health insurance, etc.; 2) disease information, including the date of diagnosis, category of TB, information about hospitalization, treatment solutions, duration of expected treatment, HIV status, etc.; 3) cost information, including hospitalization costs, examination costs, medical costs, accommodation costs, and transportation costs; and; 4) number of days lost or unemployed to patients and their caregivers due to TB.

We also collected the relevant two-level covariates of each city from the Sichuan Statistical Yearbook, including the GDP per capita, the number of health technicians and hospital beds per thousand persons, and the population density.

### Quality assurance

To ensure data quality, we adopted the following quality control measures: First, before the survey, we formulated unified survey methods and standards and all investigators from different medical institutions were trained to reduce the information bias of the investigators and improve the credibility of the conclusions. Second, as ethnic minorities account for a large proportion of the population in Sichuan Province, there may be differences in dialects. Therefore, we assigned investigators from local medical institutions to complete the questionnaire survey. Third, 5% of questionnaires were randomly selected after completing the questionnaire to check the completeness and validity. If the completeness of the questionnaire was insufficient, we conducted a supplementary survey.

### Definitions

Direct medical costs [27]: included the medical expenses required for the treatment of TB patients with TB-related symptoms before diagnosis (including outpatient registration costs, hospitalization costs, examination costs, drug costs, laboratory costs, etc.); costs related to TB diagnosis; and the costs of treatment after the diagnosis of TB.

Direct non-medical costs [28]: including transportation, accommodation and food costs required by the patient and caregivers in the process of TB care.

Direct costs = Direct medical and non-medical costs

Indirect costs [29]: loss of patient income due to TB and loss of household income due to missed work (estimated by data collection such as missed work time) of caregivers in the process of TB care. In this study, we collected the average daily wage of the patient before the diagnosis of TB and the average daily income of the household for caregivers. Because our study was from the perspective of patients or families, for patients or caregivers without wages, we treat their indirect costs as 0.

Indirect costs = number of days missed due to TB ◊ average daily income of patients and their families

Total costs = Direct costs and indirect costs

OOPE [30]: Total costs excluded insurance reimbursements and state-provided healthcare costs, including medical OOPE and non-medical OOPE paid by the patient during the TB diagnosis and treatment period.

OOPE = medical OOPE + non-medical OOPE

TOOPE: including OOPE and loss of social wealth due to sickness of patients and absence of their caregivers.

TOOPE = OOPE + indirect costs

Catastrophic total costs due to TB: total costs (indirect and direct combined) exceeding a given threshold (e.g. 20%) of the household’s annual income [31].

### Statistical analysis

We used EpiData 3.1 software to double-enter the content of the questionnaire to ensure accurate data entry. The qualitative data of this study were described by numbers and percentages (%); the measurement data subject to skewed distribution were described by medians (M) and interquartile ranges (IQRs). Univariate analysis of influencing factors used nonparametric tests. Two-sided tests were used for hypothesis testing.

Catastrophic total costs due to TB were estimated in Sichuan Province and divided into five economic zones, namely the Chengdu Plain, southern Sichuan, northeastern Sichuan, Panxi, and northwestern Sichuan regions [32]. We used the annual household incomes obtained from the questionnaire as the basis and employed imputation to fill in the missing data [31].

Since the subjects were all patients undergoing treatment, to comprehensively estimate the cost of the entire course of TB treatment, we evaluated the patients' total cost by multiplying the estimated treatment duration (in months) obtained from the survey by the cost of each visit. In order to make it easier for international readers to understand the research results, the US dollars (USD) are also expressed to the results, with the 2021 exchange rate between USD and CNY used as the basis for calculation. Because OOPE and TOOPE were skewed data, the data were log-transformed.

Considering that the data have a hierarchical structure of "city-individual", patients in the same city may not be independent because of a certain correlation. Therefore, a two-level linear regression model was used to explore the influencing factors with patients as level 1 unit and city as level 2.

First, a null model containing only the intercept term was established to determine whether level 2 was statistically significant. Then a meaningful unit-level variable was introduced as an explanatory variable. The basic structure of the model is as follows:

- Level 1 model: $${Y}_{ij}={\beta }_{0j}+{\sum }_{1}^{m}{\beta }_{m}{x}_{ij}+{e}_{ij}$$
- Level 2 model: $${\beta }_{0j}={\beta }_{0}+{\sum }_{1}^{n}{\beta }_{01}{\omega }_{nj}+{\mu }_{0j}$$
- Combined model: $${Y}_{ij}={\beta }_{0}+{\sum }_{1}^{n}{\beta }_{01}{\omega }_{nj}+{\mu }_{0j}+{\sum }_{1}^{m}{\beta }_{m}{x}_{ij}+{e}_{ij}$$

In this formula, *j* represents the city level (level 2), and *i* represents the patient level (level 1). ${Y}_{ij}$ represents the cost of the *ith* patient in the *jth* city. *m* represents *m* level 1 explanatory variables, and *n* represents *n* level 2 explanatory variables. ${\beta }_{m}$ represents the regression coefficient of explanatory variable *x* at level 1, and ${\beta }_{01}$ represents the regression coefficient of explanatory variable $\omega$ at level 2, also known as the fixed effect parameter. ${\beta }_{0}$ is the intercept; ${e}_{ij}$ is the residual at the patient level, which is the random effect of level 1. ${\mu }_{0j}$ is the residual at the city level, which is the random effect at level 2.

To avoid collinearity between covariates, Spearman's rank correlation test was used to assess associations before establishing the model (Fig. S2). In the construction of the model, all statistically significant variables without strong correlations (less than 0.6) in the univariate analysis were included in the model. The method of gradual inclusion was adopted. Random forest method was used to determine the order of variable inclusion, and the model was incorporated from large to small according to the importance (Figs. S3 and S4). Finally, the variables to be included in the model were determined according to the Akaike’s information criterion (AIC) value of the model and the practical significance of the variables.

For all the analyses, the criterion for statistical significance was α=0.05. R version 4.2.1 and IBM SPSS version 28.0 (New York, United States) statistical software were used for data analysis.

## Results

### Catastrophic total costs due to TB for patients

A total of 2644 TB patients were enrolled in this study, and 74.24% of TB patients and their households experienced catastrophic total costs due to TB. Among them, the highest incidence of catastrophic total costs due to TB was 92.31% in the northwestern Sichuan economic zone. The lowest incidence of catastrophic total costs due to TB was 72.30% in Chengdu Plain Economic Zone (Table 1).

**Table 1:** Incidence of catastrophic total costs due to TB in patients in Sichuan Province

Economic Zone	Catastrophic total costs due to TB				
	Occurrence	%	Non-occurrence	%	total
Chengdu Plain Economic Zone	736	72.30%	282	27.70%	1018
Southern Sichuan Economic Zone	598	72.57%	226	27.43%	824
Northeastern Sichuan Economic Zone	436	77.30%	128	22.70%	564
Panxi Economic Zone	169	79.72%	43	20.28%	212
Northwestern Sichuan Economic Zone	24	92.31%	2	7.69%	26
Total	1963	74.24%	681	25.76%	2644

### Socio-demographic characteristics

Among the 2644 TB patients, 91.64% were from the investigated counties, and the proportion of males (69.30%) was much higher than that of females (30.70%). Among the four age groups, the largest number of TB patients was 41~60 years old (34.14%) and almost the type of TB category (94.40%) were new cases. More than half of the patients' occupations (57.20%) were migrant workers in the service industry. Most patients had no history of hospitalization (68.09%) or other chronic diseases (65.89%). Overall, 95.12% of TB patients did not have other types of TB, and 98.87% of TB patients received the first-line anti-TB regimen (Table 2).

**Table 2:** Socio-demographic characteristics of the TB participants in Sichuan Province

Variables	Number	Percent (%)
Gender		
Male	1832	69.29
Female	812	30.71
Age (years)		
≤20	311	11.76
21~40	626	23.68
41~60	903	34.15
≥61	804	30.41
Education level		
Illiteracy	306	11.57
Primary school	884	33.43
Junior high school	782	29.58
Senior high school	379	14.34
Junior college and above	293	11.08
Marital status		
Single	651	24.62
Married	1750	66.19
Divorced	96	3.63
Widowed	147	5.56
Registered permanent residence		
Investigated counties	2423	91.64
Other counties	175	6.62
Other provinces	46	1.74
Economic Zone		
Chengdu Plain Economic Zone	1018	38.50
Southern Sichuan Economic Zone	824	31.16
Northeastern Sichuan Economic Zone	564	21.33
Panxi Economic Zone	212	8.02
Northwestern Sichuan Economic Zone	26	0.99
TB category		
New case	2496	94.4
Relapse case	148	5.60
Insurance		
No	67	2.60
Yes	2577	97.40
Occupation		
Student	299	11.31
Service personnel	319	12.07
Migrant workers	1513	57.22
Company employees	35	1.32
Retiree	148	5.60
Unemployed	216	8.17
Others	114	4.31
Family size		
<4	1570	59.38
≥4	1074	40.62
HIV		
Positive	84	3.18
Negative	1916	72.46
Unknown	644	24.36
Hospitalization		
Yes	844	31.92
No	1800	68.08
Comorbidity		
No	1799	68.05
Diabetes	299	11.31
Chronic liver disease	59	2.23
CKD	20	0.76
Anemia	55	2.08
Hypertension	215	8.13
Other	284	10.74
Number of visits before diagnosis		
<2	1494	56.51
≥2	1150	43.49
Treatment solutions		
First-line anti-TB regimen	2614	98.87
Second-line anti-TB regimen	30	1.13
Combined with other type of TB		
Yes	129	4.88
No	2515	95.12

The median GDP per capita and population density were 44140.5 (IQR: 35352.75, 57002) and 350/km^2^ (IQR: 183, 578), respectively, and the medians of the number of health technicians and hospital beds per thousand persons were 6.28 (IQR:5.60, 7.13) and 7.56 (IQR: 6.67, 8.24), respectively. The collected two-level covariates in each city are shown in Table S1.

### The costs of TB patients

The median total cost was 9223.37 CNY (1429.98 USD), in which the median direct and indirect costs of TB patients were 10185.00 CNY (1579.07 USD) and 2400.00 CNY (372.09 USD), respectively, and indirect costs contributed to 43% of total costs. The median direct medical cost was 8687.50 CNY (1346.90 USD), and the median direct non-medical cost was 1151.50 CNY (178.53 USD). The median OOPE and TOOPE costs were 6024.00 CNY (933.95 USD) and 11890.50 CNY (1843.49 USD), respectively (Table 3).

**Table 3:** The total costs of TB patients in Sichuan Province (CNY)

	Median	IQRs	Mean	SD
Direct costs	10185.00	(4741.25,19167.75)	16300.94	24905.34
Direct medical costs	8687.50	(3905.25,16750.00)	14149.30	23115.07
Direct non-medical costs	1151.50	(372.25,2622.00)	2151.64	3541.88
Food costs	500.00	(100.00,1128.75)	925.74	1661.73
Transportation costs	190.00	(76.00,423.00)	355.98	574.27
Accommodation costs	0.00	(0.00,0.00)	161.13	579.90
Other costs	200.00	(0.00,800.00)	708.79	1666.82
OOPE	6024.00	(2642.50,12210.50)	9933.75	13687.24
Indirect costs	2400.00	(0.00,14830.80)	12319.42	25740.84
Patient costs	1532.00	(0.00,14253.60)	11764.41	25459.27
Family costs	0.00	(0.00,120.00)	555.01	2053.87
TOOPE	11890.50	(4769.75,28273.50)	22253.17	31815.62
Total costs	9223.37	(7761.75,36205.25)	28620.37	38867.78

### Univariate analysis of the influencing factors of patients' costs

The univariate analysis showed that there were statistically significant differences in OOPE in age, marital status, household registration, economic zone, insurance, occupation, and family size groups (*P* < 0.05, Table 4). Meanwhile, in different gender, age, education level, marital status, household registration, economic zone, insurance, occupation, and family size groups, significant differences in TOOPE were observed (*P* < 0.05, Table 4).

**Table 4:** Univariate analysis of the influencing factors of TB patients' costs with different Socio-demographic characteristics in Sichuan Province

Variable	OOPE			TOOPE		
	M	IQRs	*P*	M	IQRs	*P*
Gender						
Male	6193.00	(2697.00,12230.75)	0.240	12888.00	(5319.25,30438.75)	<0.001
Female	5810.00	(2610.25,12161.75)		9514.00	(3876.75,24623.10)	
Age(years)						
≤20	4127.00	(1617.00,7930.00)	<0.001	4648.00	(1713.00,9500.00)	<0.001
21~40	5224.50	(2002.75,10943.50)		16160.00	(4164.75,40935.25)	
41~60	6589.00	(2934.00,13252.00)		17249.60	(7274.00,36400.00)	
≥61	7333.00	(3556.50,13228.00)		10028.50	(5097.25,5097.25)	
Education level						
Illiteracy	5340.00	(1689.50,10535.00)	0.054	7727.50	(2582.00,14858.20)	<0.001
Primary school	6161.00	(2780.75,12114.00)		11649.50	(5333.25,25595.72)	
Junior high school	6343.50	(2575.75,13001.75)		15479.10	(5668.25,33566.25)	
Senior high school	5607.00	(2655.00,12320.00)		10380.00	(4333.00,34530.00)	
Junior college and above	6078.00	(3134.00,11615.50)		12178.00	(4835.00,31935.50)	
Marital status						
Single	4915.00	(2032.00,9728.00)	<0.001	7630.00	(2655.00,20600.00)	<0.001
Married	6423.50	(2731.25,12760.00)		13238.50	(5960.45,29494.50)	
Divorced	7147.50	(3591.50,15510.25)		27240.00	(11003.50,58176.05)	
Widowed	7620.00	(4278.00,15747.00)		10598.00	(5094.00,23005.00)	
Registered permanent residence						
Investigate counties	5878.00	(2516.00,11700.00)	<0.001	11174.00	(4641.00,27086.00)	<0.001
Other counties	9317.00	(4366.00,15416.00)		20179.40	(9117.00,49614.00)	
Other provinces	7215.50	(3512.50,14527.50)		16035.00	(4745.00,37563.50)	
Economic Zone						
Chengdu Plain Economic Zone	9335.00	(4595.00,16677.25)	<0.001	17168.00	(7701.00,37723.50)	<0.001
Southern Sichuan Economic Zone	5664.50	(2627.25,10228.50)		10412.00	(4706.75,26117.50)	
Northeastern Sichuan Economic Zone	4809.00	(3010.75,8171.50)		10840.50	(5369.25,21788.75)	
Panxi Economic Zone	1111.00	(525.50,1913.75)		1232.50	(570.00,2891.25)	
Northwestern Sichuan Economic Zone	16155.00	(111120.50,35840.25)		20340.00	(14802.50,40433.25)	
Insurance						
No	8029.50	(3289.25,19714.00)	0.016	27021.00	(7649.92,58321.25)	<0.001
Yes	5961.00	(2626.00,12142.25)		11772.00	(4473.25,27762.75)	
Occupation						
Student	4316.00	(1928.00,8327.00)	<0.001	4604.00	(1940.00,9110.00)	<0.001
Service personnel	7485.00	(4110.00,14592.00)		27688.00	(12996.00,53762.00)	
Migrant workers	5632.00	(2255.50,11114.00)		12360.00	(4997.10,28456.30)	
Company employees	8396.00	(4170.00,18108.00)		18944.00	(5712.00,49379.00)	
Retiree	9616.50	(5319.25,14645.25)		10873.50	(6625.00,17529.75)	
Unemployed	7125.00	(3536.50,15347.25)		9595.50	(4850.00,20547.30)	
Others	8205.00	(2250.50,14439.50)		17981.20	(5693.75,46591.00)	
Family size						
<4	6645.50	(3019.50,12967.75)	<0.001	12485.50	(5592.85,29895.65)	<0.001
≥4	5320.00	(2039.25,10887.25)		11032.50	(3623.50,26080.90)	

In the different diagnosis and treatment situations, univariate analysis showed that there were statistically significant differences of OOPE and TOOPE in TB category, HIV, hospitalization, comorbidity, number of visits before diagnosis, treatment solutions, and combined with other type of TB groups (*P* < 0.05, Table 5).

**Table 5:** Univariate analysis of the influencing factors of TB patients' costs with differential diagnosis and treatment situations in Sichuan Province

variables	OOP expenditure			TOOPE		
	M	IQRs	*P*	M	IQRs	*P*
TB category						
New case	5953.50	(2570.50,11954.25)	0.001	11645.50	(4686.50,27910.00)	0.003
Relapse case	8558.00	(3654.25,16168.25)		15164.50	(6564.00,36688.90)	
HIV						
Positive	5470.00	(1441.25,12684.00)	<0.001	8465.00	(1740.25,34323.80)	<0.001
Negative	5591.00	(2543.50,10917.25)		10692.20	(4604.25,25996.00)	
Unknown	7874.50	(3301.50,15280.75)		16621.40	(6803.75,37388.50)	
Hospitalization						
No	5211.00	(2387.25,10010.25)	<0.001	10744.50	(4486.50,24656.85)	<0.001
Yes	8763.50	(4008.75,17422.75)		14965.30	(5851.75,37269.55)	
Comorbidity						
No	5045.00	(2128.50,9897.00)	<0.001	10161.00	(3726.50,26534.50)	<0.001
Yes	9160.00	(4620.00,15795.00)		15319.40	(7450.00,32055.00)	
Number of visits before diagnosis						
<2	4795.00	(1628.75,9510.00)	<0.001	8935.00	(2760.50,21377.25)	<0.001
≥2	8098.50	(4312.00,15417.50)		16183.50	(7694.25,35724.50)	
Treatment solutions						
First-line anti-TB regimen	5949.00	(2620.00,12049.25)	<0.001	11772.00	(4721.50,28007.50)	<0.001
Second-line anti-TB regimen	17769.00	(8022.75,24699.50)		32263.50	(12505.50,94150.60)	
Combined with other types of TB						
No	5880.00	(2551.00,11829.00)	<0.001	11452.00	(4608.00,27296.00)	<0.001
Yes	10230.00	(5586.00,22167.00)		28086.00	(9926.00,57126.00)	

### Multilevel analysis of influencing factors of TB patients' costs

We fitted the null model of the two-level linear regression, and the fixed scale parameter was set to 1. The results showed that the differences at the two-level were statistically significant (*P*<0.05), which indicated that the two-level linear regression model was appropriate in this study to estimate the influencing risk factors for OOPE and TOOPE in TB patients in Sichuan Province (Table S2).

**Fig. 1:**
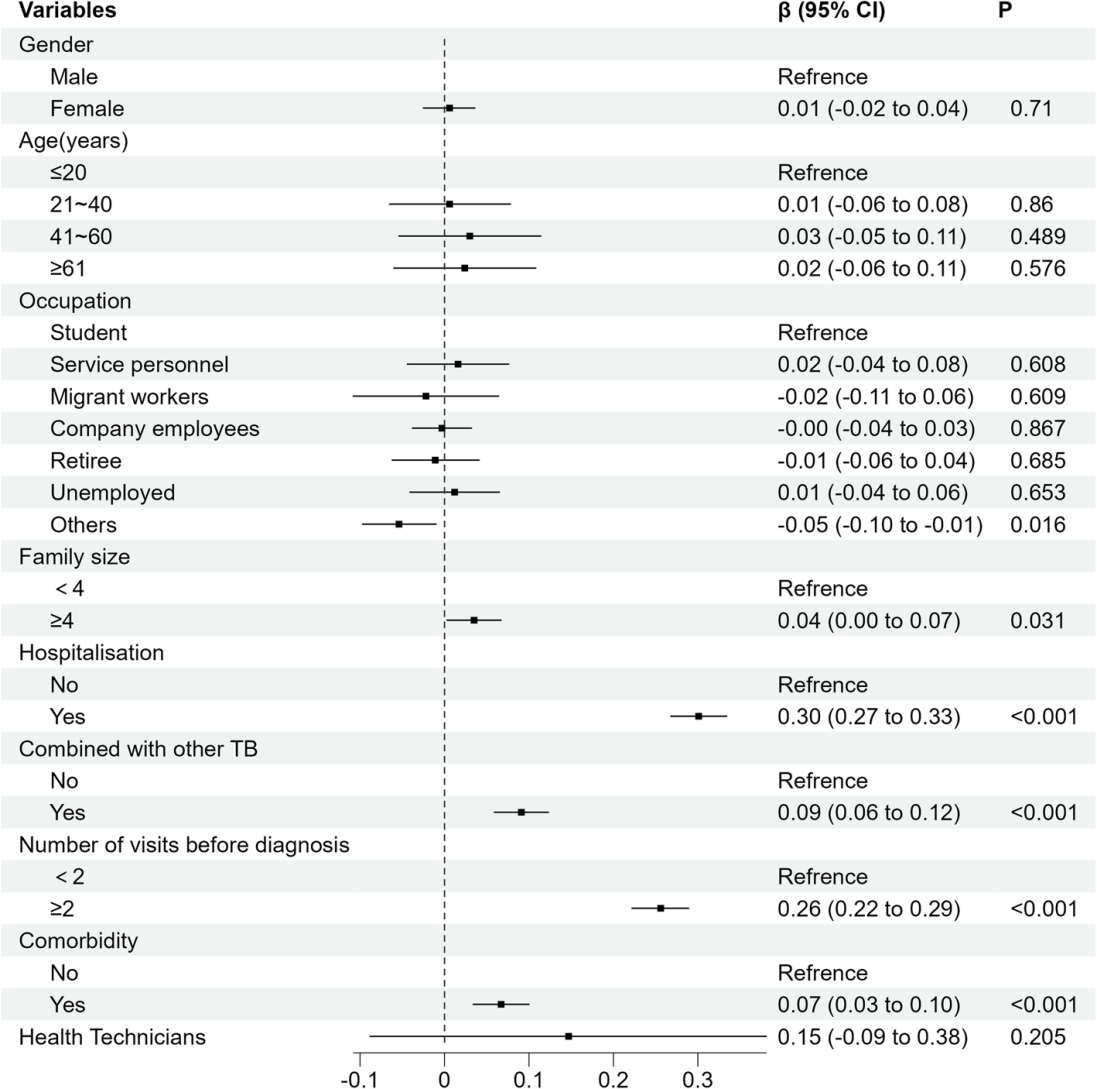
Two-level linear regression model of influencing factors of OOPE in TB patients in Sichuan Province

**Fig. 2:**
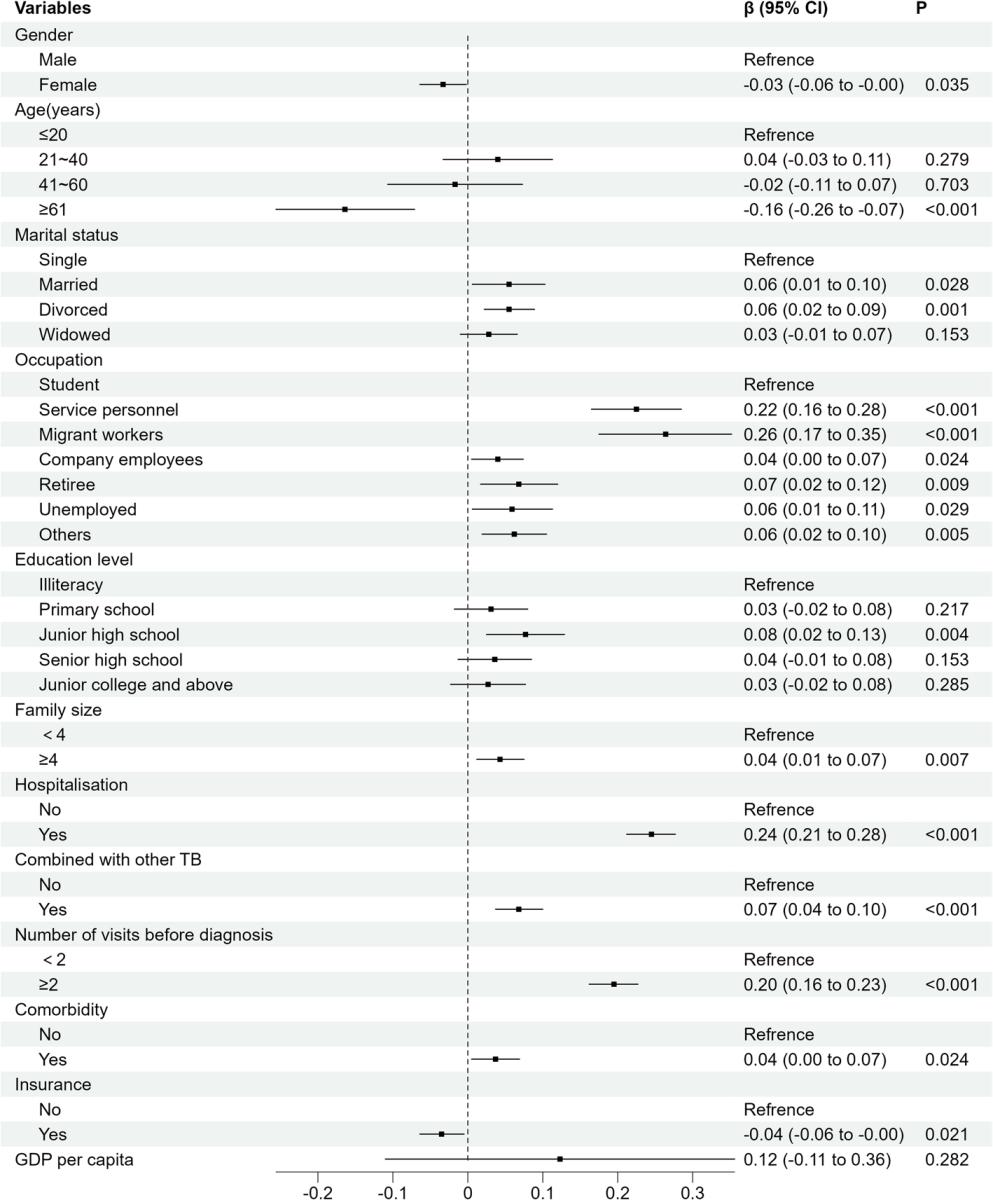
Two-level linear regression model of influencing factors of TOOPE in patients in Sichuan Province

The results of two-level linear regression showed that health technicians per capita were positively associated with OOPE, and GDP per capita was positively associated with TOOPE. The OOPE and TOOPE had common influence factors, including whether the patient's family size was ≥4, hospitalization history, combination with other types of TB, number of visits before diagnosis, and combination with chronic disease. In addition, OOPE is also influenced by whether the patient's occupation is others. Furthermore, the study also showed that TOOPE was affected by gender, age, marital status, occupation, education level, and insurance (Figs. 1 and 2).

## Discussion

This study showed that the economic burden on TB patients in Sichuan Province is still heavy and has substantial economic losses in OOPE and TOOPE. The incidence of catastrophic total costs due to TB is as high as 74.24%. Furthermore, GDP per capita and health technicians per thousand persons were found to lead to an increase in OOPE and TOOPE. In addition, family size, history of hospitalization, combination with other TB, number of visits before diagnosis ≥2 times and comorbidities were identified as the primary one-level factors associated with higher OOPE and TOOPE.

The results of this study showed that the majority of TB patients were male, and more than half were migrant workers, which was consistent with the WHO report on TB incidence published in 2020 [3]. Because men have more opportunities to go out for work and have a wide range of social activities, they are more likely to contact infected people than women. Some studies [33, 34] have shown that men have higher rates of smoking and drinking than women [33, 34]. Nicotine and alcohol in tobacco can inhibit cellular immune regulation and tumor necrosis factor-α, which may lead to a greater likelihood of progression to active TB [34]. Our study also showed that two-thirds of the TB patients included did not have combined with other diseases. Among the diseases that were frequently combined, the highest incidence was diabetes. This was closely related to the fact that diabetes is one of the major drivers of TB [6]. Nearly half of TB patients visited the doctor more than twice before being made a definite diagnosis, indicating that patients may have delayed diagnosis. This undoubtedly increases the costs of patients, especially in terms of TOOPE [35].

From the perspective of influencing factors at the city level, the higher the health technicians per capita, the higher the medical level of the city was, which indicated that patients were more likely to obtain high-level medical and health services and may increase the direct medical costs of TB patients. The higher the GDP per capita, the higher the TOOPE of patients. Because the daily wage in a city with higher GDP per capita was higher, the indirect costs of working days lost due to illness during treatment would also increase, which directly led to an increase in the patient's TOOPE.

The two-level linear regression models showed that patient's family size of ≥ 4 was a risk factor for OOPE and TOOPE and was likely to have more attendants during hospitalization [36]. During a patient's hospital stay, the cost of food and accommodation for the caregiver significantly increases, along with the indirect cost of the caregiver's absence from the work. Patients with a history of hospitalization also had higher medical and family care costs [37]. In addition, a hospitalized patient had a more severe condition, so the indirect costs increased accordingly. TB patients combined with other types of TB also led to an increase in OOPE and TOOPE. According to a South Korean study, the costs associated with TB spondylitis were found to be substantial [38]. Undoubtedly, having other types of TB leads to higher direct medical costs, such as drug and testing costs.

The greater the number of visits before diagnosis, the higher the OOPE and TOOPE cost incurred. It is important to note that the measures for Tuberculosis Control and Administration of Sichuan Province [39] involve centralized management and treatment of TB patients within the province. Suspected TB patients who are not yet diagnosed and are identified by urban subdistrict or village health organizations should be registered and transferred to designated TB medical institutions for treatment. For diagnosed patients, chemotherapy should be supervised throughout the treatment course to ensure the completeness of the patient's treatment. However, due to the specific geographical location and administrative jurisdiction of Sichuan Province, a majority of patients belong to ethnic minority groups. The social and economic status of patients in these areas is relatively poor, with a generally low level of education that results in a lack of awareness about disease prevention and treatment, ultimately affecting their access to medical and regular treatment [40]. It is not surprising that when the patient is undiagnosed, each visit to the hospital can result in high medical examination costs and even misdiagnosed treatment costs, most of which are not covered by basic medical insurance [21]. Obviously, the increased number of visits before diagnosis greatly increases the costs of TB patients.

When TB is combined with other chronic diseases, it also increases costs. For example, diabetes and anemia, risk factors for the development of TB [41], also result in significant costs for TB patients [42]. At the same time, there are also studies in the United States that the economic burden of chronic liver disease to patients should not be underestimated [8]. The economic burden of patients with CKD in Spain studied by Escobar et al [43] showed that the cumulative costs associated with CKD in patients with CKD reached €14,728.4 over a 5-year period. This shows that patients with CKD have an economic burden that cannot be ignored. When patients have both TB and CKD, the economic burden is undoubtedly heavier.

Our study indicated that patients might have less TOOPE when they were female or older than 61 years of age. Part of the reason may be that women or older patients are more frugal, so they may have less to spend on nutrition. This may also be partly due to the fact that women or older patients earned less than men or the younger, and thus have less indirect costs to lose from treatment during treatment. The TOOPE was higher in married and divorced patients than in single patients. On the one hand, it may be that married patients and divorced patients were mostly older, and their wages were higher than those of younger patients so that the indirect costs will increase. On the other hand, according to the research of Huang Y, the cost of divorced patients during diagnosis is higher [44]. Our study found that patients with occupations had lower OOPE than students because students are growing, especially during illness, when diet and nutrition can be more expensive. Consequently, patients in other occupations experience lower direct non-medical costs, including food and drink costs, during their outpatient and inpatient stays. With regards to TOOPE, patients with formal occupations had higher TOOPE than students. This can be explained by the fact that students do not lose working days due to TB during treatment, resulting in lower indirect costs compared to other occupations. Additionally, TOOPE was also affected by the patient's level of education. Compared with illiterate patients, the economic costs of patients in junior high school were higher. When patients receive only junior middle school education, they will be more aware of seeing a doctor and pay more attention to health than illiterate patients [45]. However, compared with patients with higher education, patients with junior middle school education may not better understand hospital procedures and medical knowledge [46]. As a result, patients with a junior high school education may spend more money before diagnosis and during treatment.

In summary, the results of this study show that, firstly, patients with a history of hospitalization and extra-pulmonary TB will have increased the OOPE and TOOPE. Relevant institutions should strengthen the publicity and education of TB related knowledge, popularize basic medical knowledge to the public, improve the public's awareness of medical treatment and early treatment, and avoid the aggravation of illness and hospitalization due to complications caused by delay so as to reduce the subsequent economic costs. Secondly, the OOPE and TOOPE of patients will also increase due to the increase in the number of visits before diagnosis. Therefore, it is necessary to improve the service system of medical institutions, improve the sensitivity of TB diagnosis, and allow patients to be diagnosed once, so as to reduce the unnecessary economic expenditure of patients. Finally, the combination of chronic diseases also increases the cost of treatment for patients. We should strengthen the knowledge of chronic diseases, raise public awareness of health examinations, and control chronic diseases as soon as possible.

Our study had some limitations. Firstly, we selected TB patients attending each institution to reduce survey bias. However, when patients recall the time spent before diagnosis, there may still be recall bias because of time. Secondly, the doctor's treatment plan calculates the cost at the end of treatment. Although there is some regularity in the frequency and dose of visits during the treatment phase, it is reasonable to use the doctor's follow-up treatment plan to estimate costs. However, this approach may ignore the uncertain costs patients may incur in future treatment. Finally, our study included only patients with drug-sensitive TB, not drug-resistant TB. Caution should be exercised in generalizing the results of this study to include other groups of patients with drug-resistant TB.

## Conclusions

Despite China's policy of providing free treatment for TB patients and a series of safeguard measures, we found that OOPE and TOOPE for TB patients in Sichuan Province continued to remain high. In addition to sociological factors, family size, history of hospitalization, extra-pulmonary TB, number of visits before diagnosis ≥2 times, and comorbidities are all indirect factors that cannot be ignored. Effective policies and measures could reduce the costs of patients and bring great economic benefits to the country and society. It is imperative to enhance public awareness and education on TB prevention and control, thereby promoting early detection and treatment of TB and suspected TB infections at designated medical institutions, ultimately reducing the associated costs. At the same time, the government should introduce more policies to benefit the people, increase the reimbursement of expenses related to hospital treatment for patients, and appropriately increase the direct non-medical expense subsidies for patients, so as to reduce the treatment cost of patients. Overall, the findings of our study provide valuable insights for reducing the costs of TB patients, which can provide a basis for the government and related institutions to develop and enhance policies aimed at addressing these challenges, as well as provide some clues for further research in this field.

## Supplementary Information

**Additional file 1.**

## Data Availability

The data presented in this study are available on reasonable request from the corresponding author.
