# Supplementary material for: Assessing the influencing factors of out-of-pocket costs on tuberculosis in Sichuan Province: a cross-sectional study
Source: BMC Public Health. 2023 Jul 19;23:1391. doi: 10.1186/s12889-023-16180-y (PMC10357819; doi:10.1186/s12889-023-16180-y)
Supplement: Supplementary file 1 — Additional file 1. [file 12889_2023_16180_MOESM1_ESM.docx]

***Supplementary materials***

**Assessing the influence factors of Out-of-Pocket Costs on tuberculosis in Sichuan province: A Cross-Sectional Study**


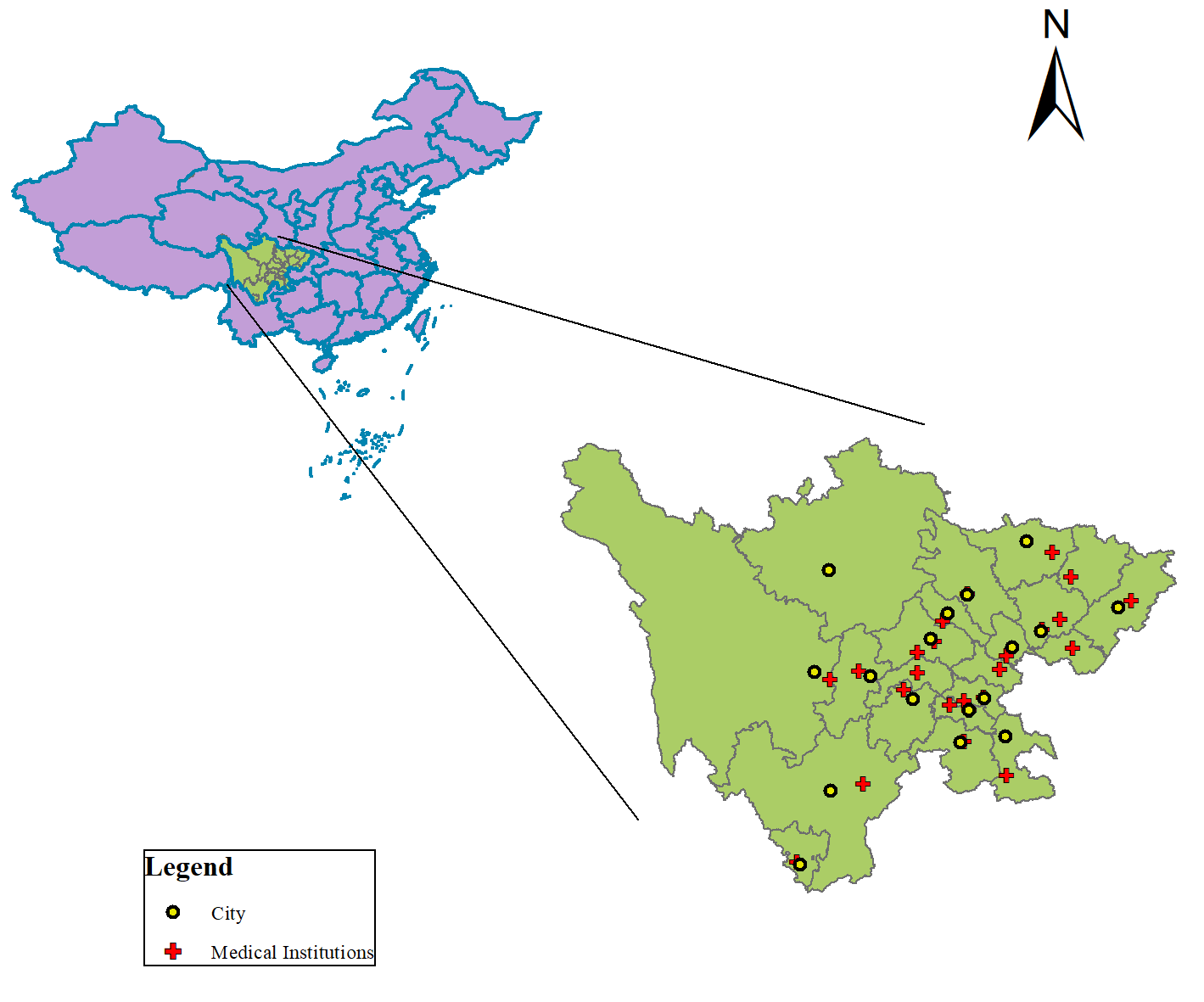


**Figure S1.** Distribution map of 25 designated medical institutions in Sichuan Province


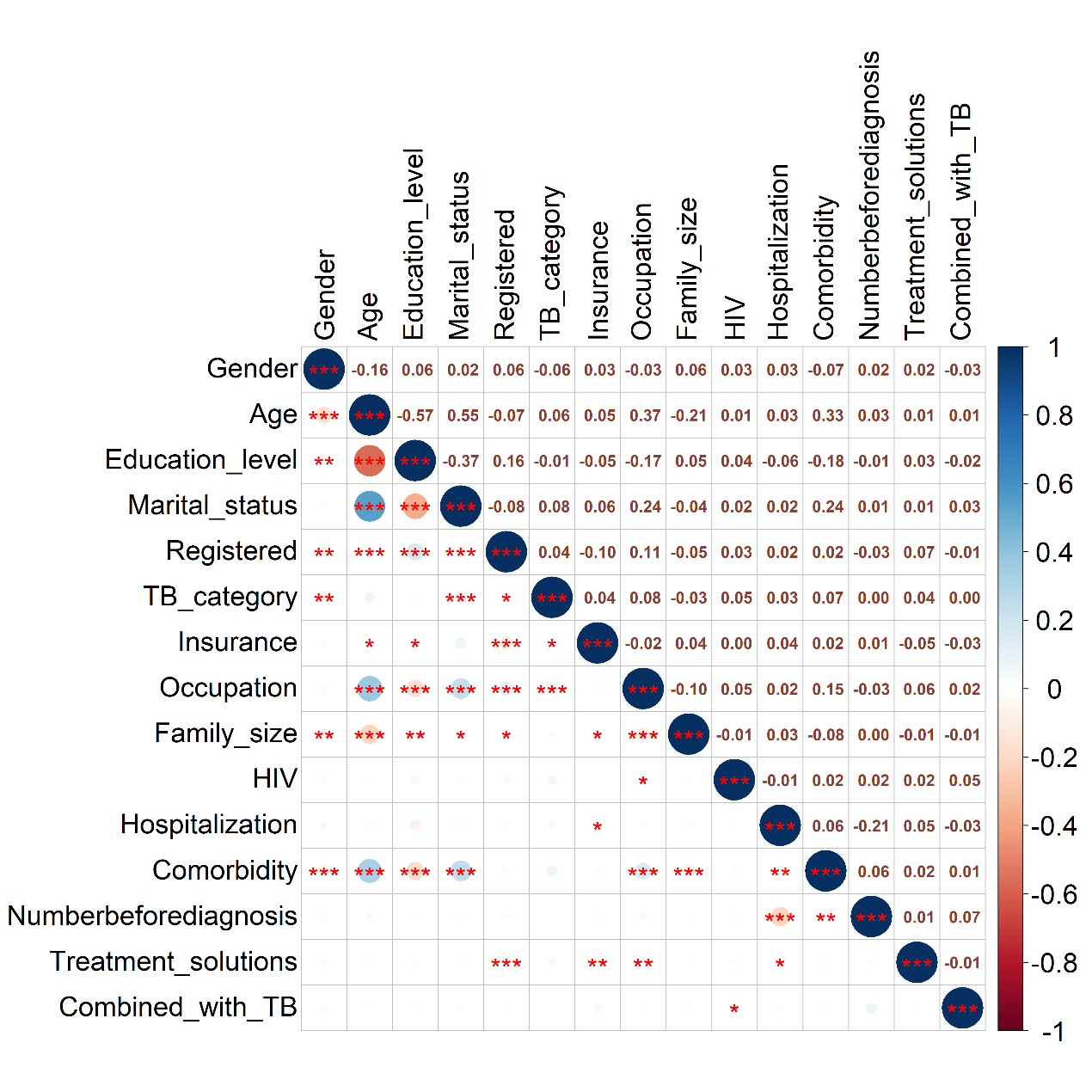


**Figure S2.** The Spearman correlation diagram between covariates in Sichuan Province


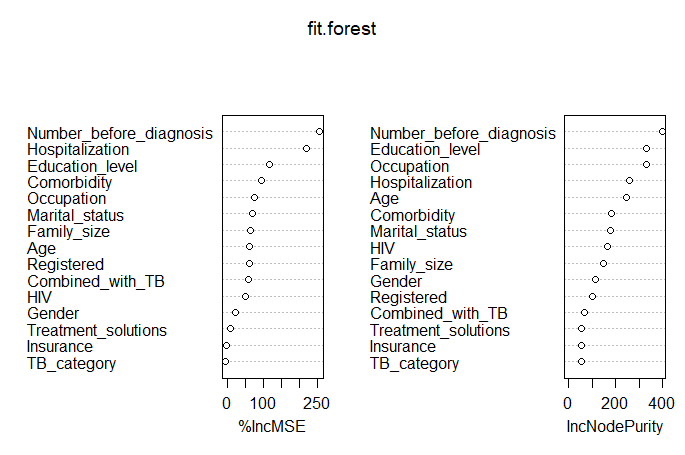


**Figure S3.** The random forest of OOPE


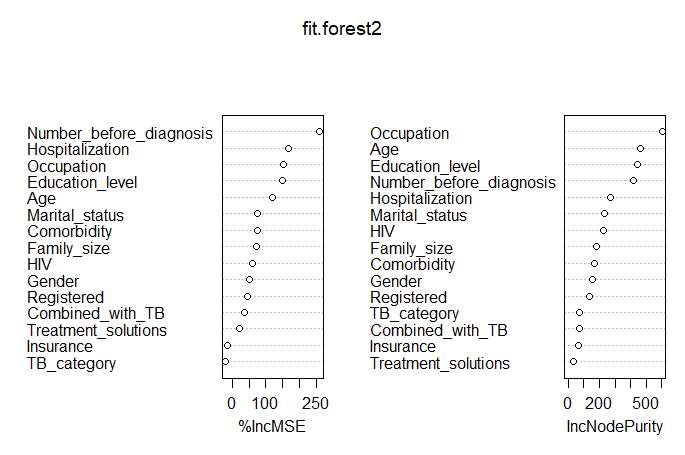


**Figure S4**. The random forest of TOOPE

**Table S1** The covariates of two-level variables in each city in Sichuan Province

| City | GDP per capita | Health technicians (per thousand people) | Hospital beds (per thousand people) | Population density (km^2^) |
| --- | --- | --- | --- | --- |
| Bazhong | 22716 | 5.34 | 6.82 | 270 |
| Chengdu | 103386 | 11.15 | 8.98 | 1157 |
| Dazhou | 35625 | 4.88 | 6.25 | 346 |
| Deyang | 65745 | 6.95 | 7.40 | 603 |
| Ganzizhou | 32440 | 5.65 | 4.49 | 8 |
| Guangan | 38522 | 5.28 | 6.55 | 513 |
| Guangyuan | 35262 | 7.15 | 8.93 | 164 |
| Leshan | 56999 | 6.70 | 7.74 | 257 |
| Liangshanzhou | 34085 | 5.57 | 5.71 | 82 |
| Luzhou | 48105 | 7.07 | 7.98 | 354 |
| Meishan | 46168 | 6.04 | 6.66 | 419 |
| Mianyang | 58685 | 7.17 | 8.27 | 241 |
| Nanchong | 36073 | 5.58 | 6.80 | 516 |
| Neijiang | 38743 | 5.70 | 6.99 | 687 |
| Panzhihua | 82460 | 8.91 | 8.61 | 164 |
| Suining | 42113 | 5.72 | 6.71 | 599 |
| Yaan | 46984 | 8.33 | 8.59 | 102 |
| Yibin | 57003 | 6.52 | 7.71 | 345 |
| Ziyang | 31019 | 5.97 | 8.08 | 436 |
| Zigong | 48904 | 6.99 | 8.14 | 667 |

**Table S2.** Null model of the two-level linear regression

| Variables | Estimate | Std. Error | *t/Z* | *P* |
| --- | --- | --- | --- | --- |
| **OOPE** | |  |  |  |
| Fixed effects |  |  |  |  |
| Intercept | 8.706 | 0.150 | 58.049 | ＜0.001 |
| Random effects | |  |  |  |
| level 2 | 0.434 | 0.145 | 2.991 | 0.003 |
| level 1 | 1.240 | 0.034 | 36.223 | ＜0.001 |
| ICC* | 0.259 |  |  |  |
| **TOOPE** | |  |  |  |
| Fixed effects | 9.373 | 0.166 | 56.599 | ＜0.001 |
| Intercept |  |  |  |  |
| Random effects | |  |  |  |
| level 2 | 0.529 | 0.176 | 3.010 | 0.003 |
| level 1 | 1.553 | 0.043 | 36.225 | ＜0.001 |
| ICC* | 0.254 |  |  |  |

Intro-class correlation coefficient (ICC) *: ICC value indicates the proportion of the total variation that multi-level variables can explain, and the value ranges from 0 to 1. The larger the ICC value is, the more part can be explained by two-level variables, so multi-level model analysis should be considered; OOPE: Out-of-pocket expenditure; TOOPE: Total out-of-pocket expenditure.
